# Prevalence of BRCA homopolymeric indels in an ION Torrent-based tumour-to-germline testing workflow in high-grade ovarian carcinoma

**DOI:** 10.1038/s41598-023-33857-x

**Published:** 2023-05-13

**Authors:** Jacopo Azzollini, Luca Agnelli, Elena Conca, Tommaso Torelli, Adele Busico, Iolanda Capone, Marta Angelini, Elena Tamborini, Federica Perrone, Andrea Vingiani, Daniele Lorenzini, Bernard Peissel, Giancarlo Pruneri, Siranoush Manoukian

**Affiliations:** 1 Unit of Medical Genetics, Department of Medical Oncology and Hematology, Fondazione IRCCS Istituto Nazionale dei Tumori, via Venezian 1, 20133 Milan, Italy; 2 Department of Advanced Diagnostics, Fondazione IRCCS Istituto Nazionale dei Tumori, via Venezian 1, 20133 Milan, Italy; 3 Medical Oncology 1 Department, Fondazione IRCCS Istituto Nazionale dei Tumori, via Venezian 1, 20133 Milan, Italy; 4 Oncology and Hemato-Oncology Department, University of Milan, Milan, Italy

**Keywords:** Clinical genetics, Cancer genetics, Ovarian cancer, Next-generation sequencing

## Abstract

Tumour DNA sequencing is essential for precision medicine since it guides therapeutic decisions but also fosters the identification of patients who may benefit from germline testing. Notwithstanding, the tumour-to-germline testing workflow presents a few caveats. The low sensitivity for indels at loci with sequences of identical bases (homopolymers) of ion semiconductor-based sequencing techniques represents a well-known limitation, but the prevalence of indels overlooked by these techniques in high-risk populations has not been investigated. In our study, we addressed this issue at the homopolymeric regions of BRCA1/2 in a retrospectively selected cohort of 157 patients affected with high-grade ovarian cancer and negative at tumour testing by ION Torrent sequencing. Variant allele frequency (VAF) of indels at each of the 29 investigated homopolymers was systematically revised with the IGV software. Thresholds to discriminate putative germline variants were defined by scaling the VAF to a normal distribution and calculating the outliers that exceeded the mean + 3 median-adjusted deviations of a control population. Sanger sequencing of the outliers confirmed the occurrence of only one of the five putative indels in both tumour and blood from a patient with a family history of breast cancer. Our results indicated that the prevalence of homopolymeric indels overlooked by ion semiconductor techniques is seemingly low. A careful evaluation of clinical and family history data would further help minimise this technique-bound limitation, highlighting cases in which a deeper look at these regions would be recommended.

## Introduction

Tumour genomic profiling has a central role in the diagnosis, drug response prediction and prognosis of cancer patients^1–3^. Simultaneous sequencing of the full coding regions of multiple genes by next-generation sequencing (NGS) techniques^4^ allows the identification of a high number of variants, including putative germline variants in cancer-predisposing genes, to be actioned by targeted therapies and/or *ad-hoc* available preventive options (i.e. intensive surveillance and/or prophylactic surgery)^5,6^.

Following the approval of therapies targeting homologous recombination defects (HRD) in several cancer settings, tumour testing for the Hereditary Breast and Ovarian Cancer Syndrome (HBOC) genes *BRCA1* and *BRCA2* has rapidly spread^7,8^. Although some Centres perform parallel tumour and germline testing, tumour-only sequencing followed by germline testing in selected cases is currently a clinical standard^9–11^. In this context, the management of tumour-testing results by Molecular Tumour Boards (MTBs), which are multidisciplinary teams involving clinical geneticists, has effectively improved the referral of eligible patients to genetic counselling and the detection of actionable germline variants^12^. Moreover, this multidisciplinary approach proved effective also when applied to tumours for which clinical, molecular, pathology and immunohistochemistry data have to be integrated, as in the case of colorectal cancer^13^. In our previous study, we showed that the tumour-to-germline approach could detect and overcome potential technical limitations of tumour sample analysis in identifying putative germline variants^14^, which may lead to low sensitivity for large genomic rearrangements detection, misinterpretation of secondary “reverse” mutations or reduced sequencing accuracy at loci with homopolymeric repeats.

Ion semiconductor-based sequencing is commonly used to investigate *BRCA1/2* variants in tumours, although it is known to be poorly efficient in assessing the exact length of homopolymeric stretches^15^. This flaw might yield false negative results in samples harbouring pathogenic indels: in our previous study, tumour sequencing of one ovarian cancer patient failed to identify a germline pathogenic *BRCA2* deletion at a homopolymeric region. Although this technique-bound flaw has been consistently reported, the prevalence and potential clinical relevance of miscalled BRCA variants in specific populations at increased genetic risk have not been systematically addressed^16–18^. Moreover, a deeper analysis of real-world data may be helpful in defining variant thresholds. To estimate the actual prevalence of pathogenic/likely pathogenic germline indels, we carried out a systematic analysis of *BRCA1* and *BRCA2* tumour sequencing data at homopolymeric stretches, in a cohort of consecutive high-grade ovarian cancer (HGOC) patients.

## Results

### Tumour and germline testing results

**Figure 1:**
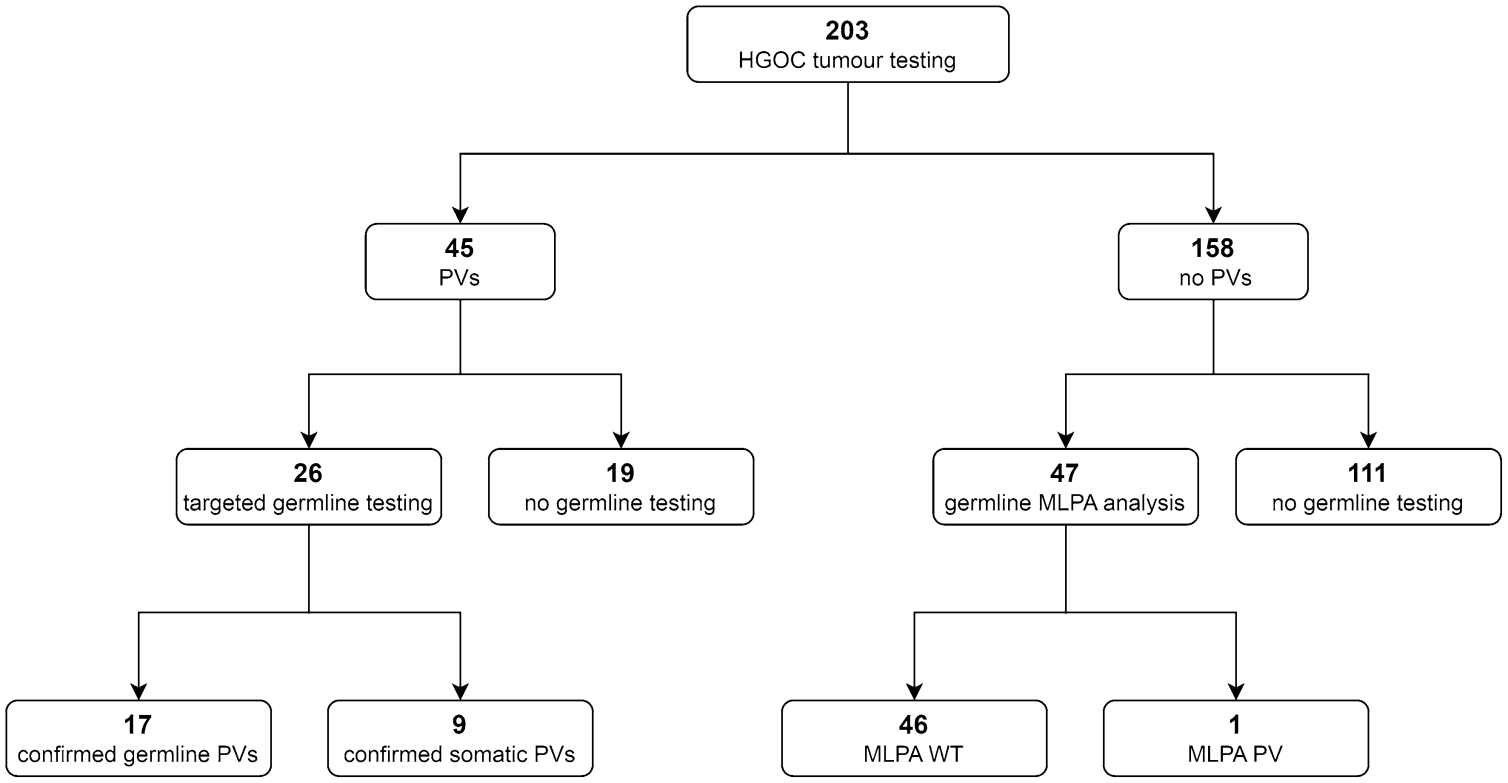
*BRCA1/2* testing results in our tumour-to-germline analysis workflow in a cohort of patients with high-grade ovarian carcinoma (HGOC). *PVs* pathogenic/likely pathogenic variants, *MLPA* multiplex ligation-dependent probe amplification, *WT* wild type.

In our cohort, which included 203 HGOC women who had not undergone prior germline analyses, 45 patients showed *BRCA1/2* pathogenic or likely pathogenic variants (henceforth termed as PVs) at tumour testing (31 *BRCA1*, 14 *BRCA2*). Twenty-six patients were subjected to genetic counselling and targeted germline testing, which identified 17 women (65%) as germline carriers and confirmed the PVs to be somatic in the remaining nine patients (35%). Among the 158 patients with no PVs detected in the tumour, 47 were offered genetic counselling with germline testing for *BRCA1/2* large rearrangements on the basis of personal or family history suggestive of HBOC, and one patient was found to bear a pathogenic out-of-frame duplication of *BRCA1* exon 13 (Fig. 1).

### Analysis of homopolymeric regions

**Figure 2:**
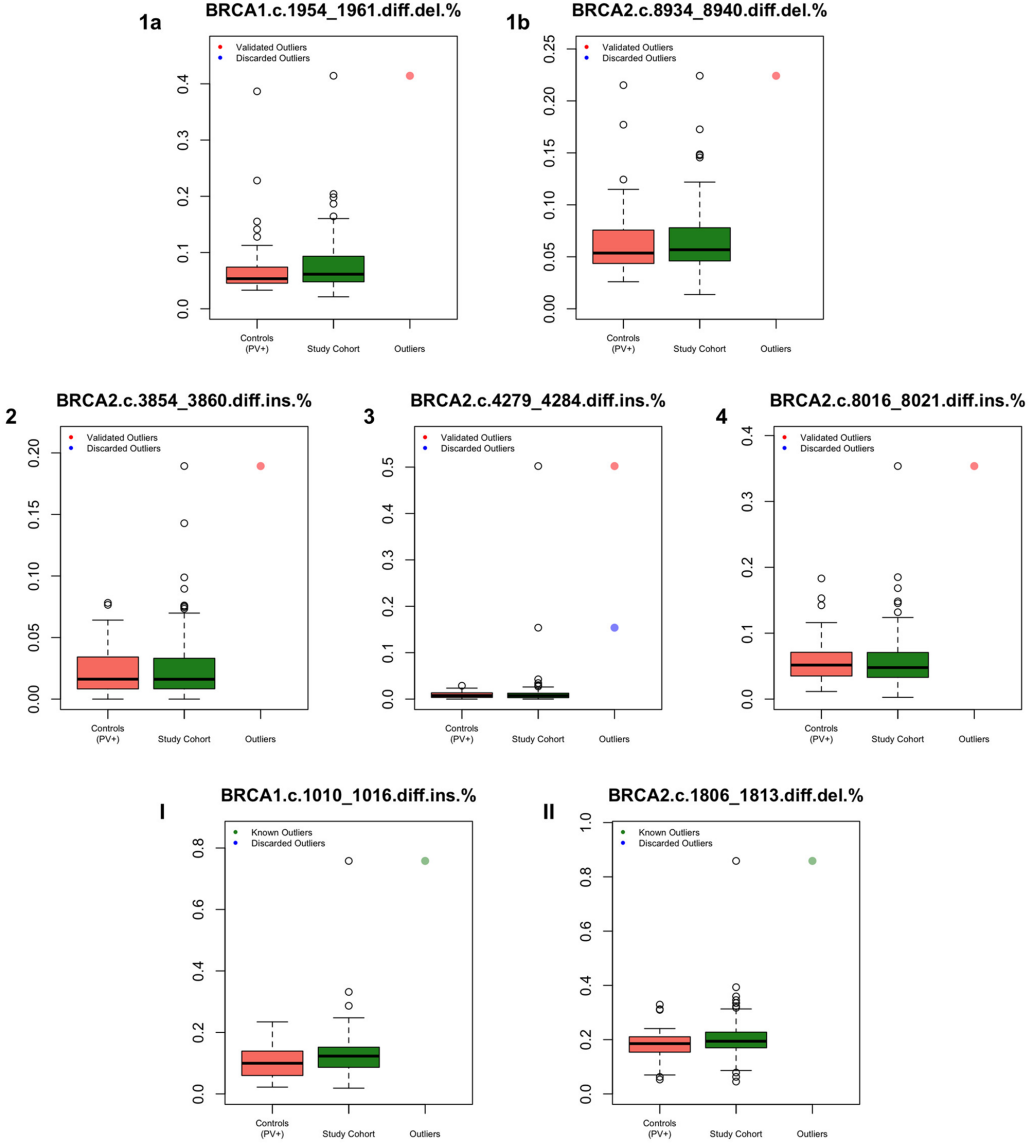
Boxplots showing the allele frequencies at tumour testing of duplications (ins) or deletions (del) at homopolymeric regions in patients with extra-homopolymer pathogenic/likely pathogenic variants (PVs, red boxes) compared with patients from the study cohort (green boxes); panels from 1 to 4 show outlier variants identified in the four patients from the study cohort (red dots), panels I and II show the two homopolymeric variants previously identified through germline testing (green dots).

Since previous studies showed that the accuracy of ion semiconductor sequencing techniques is consistently reduced at homopolymers of six or more repeated bases, we selected for the study all the homopolymeric stretches longer than five bases, including five regions at *BRCA1* and 24 at *BRCA2*. For all 203 patients included in the study, the 29 homopolymeric regions were manually visualised through the IGV software, and data on total, inserted and deleted read counts were collected. To define variant allele frequency (VAF) thresholds for homopolymeric indels, we scaled the VAF to a normal distribution and selected the outliers among the study cohort of 157 PV-negative individuals that exceeded the mean + 3 median-adjusted deviations (μ + 3σ) as compared to the normalised distribution of the VAF in the 46 carriers of non-homopolymeric PVs. These latter patients had an exceedingly low probability of carrying a second PV in these regions and were thus considered the control population. In addition, we excluded outliers with an absolute VAF below 15% and/or lower than the maximum value of the control population and/or in a region with a read count < 100X. This approach allowed us to identify two deletions (1 *BRCA1*, 1 *BRCA2*) and three duplications (*BRCA2*) in four patients, which exceeded the defined thresholds and resulted validated outliers (Fig. 2 panels 1a, 1b, 2, 3 and 4).

**Figure 3:**
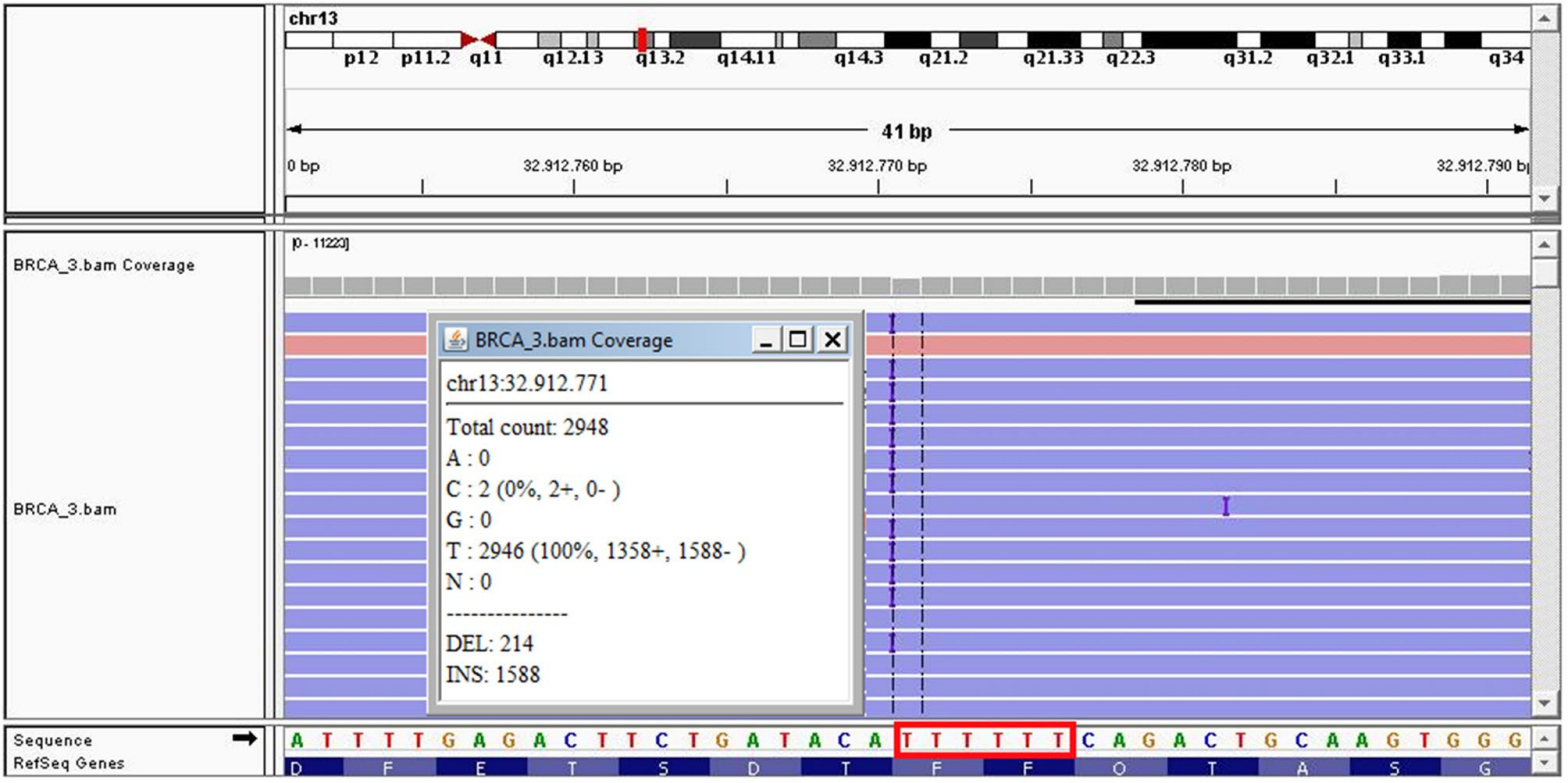
BRCA2 homopolymeric region c.4279_4284 from patient 3 visualised with the IGV software (ver. 2.3.97); the count of the total, inserted (INS) and deleted (DEL) reads at the first base of the thymine stretch (highlighted with a red rectangle) are shown in the box.

The allele frequency of the outlier homopolymer variants ranged between 22.4% and 41.4% for deletions and between 18.9 and 50.2% for duplications. Figure 3 shows the IGV software visualisation of the BRCA2 c.4279_4284 region from patient 3, displaying the homopolymeric variant with the highest allele frequency among the study cohort (Fig. 3).

Targeted Sanger sequencing on tumour DNA was performed for the five outliers to confirm the occurrence of each variant. Only the *BRCA2* c.4284dup p. (Gln1429Serfs*9) variant was confirmed at Sanger sequencing, with an allele ratio of 80%. The patient reported a positive family history (sister with breast cancer at the age of 42 years) and was one of the 47 women who had undergone genetic counselling and germline testing for large *BRCA1/2* rearrangements, which yielded negative results. The remaining four variants were not confirmed at Sanger sequencing in the tumour and were therefore considered false positive calls (Table 1).

**Table 1:** Patients with BRCA homopolymeric indels with allele frequency above the thresholds as compared with the control population; patients 1–4 were validated outliers from the study cohort, patients I and II were first identified through germline testing.

Patient #	Age at diagnosis (y)	Analysed tissue	Tumour cellularity	Outlier variants	Allele frequency in tumour	Confirmed at sanger sequencing in tumour and germline
1	78	Primary tumour (post-CT)	20%	*BRCA1* c.1961del p. (Lys654Serfs*47)	41.43%	No
				*BRCA2* c.8940del p. (Glu2981Lysfs*7)	22.42%	No
2	33	Relapse	80%	*BRCA2* c.3860dup p. (Asn1287Lysfs*2)	18.93%	No
3	62	Primary tumour	80%	*BRCA2* c.4284dup p. (Gln1429Serfs*9)	50.22%	Yes (germline)
4	55	Primary tumour	70%	BRCA2 c.8021dup p. (Ile2675Aspfs*6)	35.37%	No
I	44	Relapse	80%	*BRCA1* c.1016dup p. (Val340Glyfs*6)	75.80%	Yes (germline)
II	50	Primary tumour	70%	*BRCA2* c.1813del p. (Ile605Tyrfs*9)	85.87%	Yes (germline)

*CT* chemotherapy.

Since two samples (cases 61 and 137) failed to reach a 100X sequencing depth in nine and 16 of the 29 regions, respectively, we did not consider these as homopolymeric PV-negative cases. The estimated prevalence of homopolymeric indels in our cohort of consecutive HGOC patients was thus 0.6% (1/155). Targeted germline testing, through Sanger sequencing, of the *BRCA2* outlier duplication identified in our analysis confirmed its germline origin.

We evaluated homopolymeric indels VAFs in additional 19 patients, who were not included in the study cohort to avoid potential selection bias since germline testing was performed before tumour sequencing. Two patients were known carriers of germline homopolymeric indels, which were overlooked by the ION reporter software at tumour testing. Notably, our approach would have classified these two PVs as validated outliers (Fig. 2, panels I and II; Table 1). This finding confirmed that the thresholds defined in this study were effective in identifying actual germline homopolymeric PVs, but also pointed out that all true PVs showed a tumour VAF of 50% or more.

## Discussion

In a global scenario of steadily increasing demand for tumour molecular profiling, which has become essential to inform and guide the management of patients with cancer, tumour sequencing has been proven effective also for the identification and selection of individuals who may benefit from genetic counselling and germline testing^11,19–21^. Although this group represents only a relatively small fraction of all cancer-affected patients, the identification of germline PVs has considerable implications not only for the therapeutic and clinical management of the disease but also for the prevention of subsequent tumours and risk assessment in family members. This issue is particularly relevant in HGOC patients, in whom the prevalence of germline *BRCA1/2* PVs is estimated to be as high as 10–15%^10,22,23^, but it could also affect other BRCA-associated tumours, including advanced prostate cancer^24^.

To implement the tumour-to-germline testing model is thus crucial to address its potential limitations related to the pre-analytical process or sequencing methods used for the analysis. In our previous study, carried out in the context of our Institutional MTB, the integration of tumour and germline sequencing data, with clinical and family history information, allowed us to identify a few potential flaws. In particular, we found that among four HGOC patients, who underwent upfront germline testing and subsequent tumour sequencing, one showed discordant results, with a germline one-base deletion at a homopolymeric site of *BRCA2* that was not identified by the ION Reporter software at tumour testing.

Several reports described this limitation at *BRCA1/2* and other loci and investigated the performance of variant calling methods at homopolymers^17,25–28^. Although, as reported by Deshpande and colleagues, the ION reporter software showed an acceptable individual performance compared with other variant callers, all the studies consistently indicated a reduced sensitivity for homopolymeric variants with any variant calling method, even when used in combination.

Among 19 patients who underwent genetic counselling and germline testing, due to significant personal and family history, followed by tumour sequencing for therapeutic purposes, we previously identified two germline homopolymeric PVs, which were not reported at tumour testing by the ION reporter software (v5.6). This observation prompted us to further investigate this aspect. Albeit the low accuracy at long homopolymeric stretches represents a well-established issue, which might hinder the correct interpretation of tumour sequencing results, the frequency of *BRCA1/2* homopolymeric variant calling errors in specific cohorts of patients has never been systematically addressed. To this aim, we focused on a cohort of patients affected with HGOC, who are individuals with a higher a priori probability of being germline carriers compared with patients affected with other tumours.

We thus investigated ION Torrent sequencing data from a cohort of 203 consecutive HGOCs. Statistical thresholds for the selection of outlier variants to be investigated with an alternative technique (i.e. Sanger sequencing) were defined using allele frequencies of homopolymeric indels from 46 patients with identified extra-homopolymer PVs, who had an exceedingly low probability of carrying an additional PV at these regions. This approach, employed to select variants with the highest probability of being germline, allowed us to restrict to five the variants to be investigated. Out of 157 patients, who had resulted negative for PVs at tumour testing, only one (0.6%) was positive for a *BRCA2* duplication both in the tumour and germline. Notably, also this patient reported a family history suggestive of HBOC since her sister had developed breast cancer at the age of 42 years.

Based on our data, we expect the occurrence of *BRCA1/2* variants, which are overlooked by ION Torrent sequencing at long homopolymeric stretches, to be infrequent in HGOC and other settings. In this light, a modification of the current workflow, with a systematic and thorough assessment of homopolymeric regions, would likely result in a limited impact on the management of these patients and would not be recommended in all cases. However, it might be appropriate to consider a deeper look at these regions in patients testing negative at tumour sequencing yet reporting a personal or family history suggestive of HBOC.

There are two potential limitations to this study. First, we could not sequence all the homopolymeric regions with an alternative method for excluding the occurrence of additional homopolymeric indels. However, our approach to the definition of statistical thresholds, aimed at the detection of outlier variants, was in line with a previously published study^29^ and designed to be conservative by maintaining a high sensitivity. Based on these considerations, it seems unlikely that our approach would have overlooked true variants. This is also supported by the fact that the only confirmed homopolymeric variant was the single outlier with an allele frequency above 50%, as were the other two previously identified through germline testing. The second limitation would be that our study might have overlooked variants in homopolymers shorter than six nucleotides. The sequencing accuracy of ion semiconductor techniques has been shown to decrease for stretches longer than four consecutive identical nucleotides. However previous reports consistently reported that this flaw is more prominent as the length increases and that the accuracy steeply drops for homopolymers longer than five repetitions^15,17,25,28,30^.

In conclusion, our study provides evidence that tumour sequencing through ION Torrent is a reliable tool for detecting *BRCA1/2* germline PVs. Germline homopolymeric indels, which were overlooked by tumour sequencing, seem to be rare in HGOC and have been found only in patients with a high a priori risk of being carriers. To minimise this inherent limitation of ion semiconductor sequencing and identify patients in whom additional analyses might be indicated, a multidisciplinary approach in the context of an MTB would be preferable for better integration of molecular, clinical and family history data.

## Methods

### Patients cohort

Among consecutive patients who underwent BRCA tumour testing through ION Torrent-based sequencing between August 2017 and February 2022, we retrospectively selected 222 high-grade ovarian cancer (HGOC) patients with the following histological subtypes: 203 serous (HGSOC), seven endometrioid, five clear-cell and seven with mixed histotypes.

Since NGS *BRCA1/2* tumour testing was not available before 2017 in our Institution, 19 of 222 subjects underwent germline testing before tumour sequencing based on personal (very early age at diagnosis/previous breast cancer) or family history. According to the workflow used by our Molecular Tumour Board (MTB), in 73 out of 203 patients with upfront tumour testing subsequently received genetic counselling, either for targeted germline sequencing of a tumour-detected PV or for large genomic rearrangement analysis^14^.

The Ethics committee of Fondazione IRCCS Istituto Nazionale dei Tumori of Milan approved the use of both clinical and molecular data collected by the MTB for clinical studies and granted exemption from requiring written consent for tumour genetic testing from the patients, as these analyses were carried out in the context of a diagnostic and care setting (Approval Number INT 227/20). All the probands who underwent germline testing were aged over 18 and provided signed informed consent for the use of their biological samples and data for both diagnostic and research purposes. All methods were carried out in accordance with relevant guidelines and with the ethical principles of the Declaration of Helsinki.

### Tumour testing

The *BRCA1* and *BRCA2* genes were assessed by in-house NGS testing using the Oncomine BRCA Research Assay (Thermo Fisher Scientific, Inc). This assay provided a 100% coverage of all *BRCA1* and *BRCA2* exons, with an average of 64 bases of intronic flanking sequences upstream and downstream of each exon. Five μm sections from formalinfixed paraffin-embedded (FFPE) samples were manually microdissected to isolate the highest percentage of neoplastic cells. Genomic DNA was extracted with protease K (incubation ON at 55 °C) and quantified with Qubit dsDNA BR kit (Thermo Fisher Scientific, Inc). The libraries were prepared with the IonAmpliSeq Library kit 2.0 (Thermo Fisher Scientific, Inc) and quantified with Qubit dsDNA HS kit (Thermo Fisher Scientific, Inc) following the manufacturer’s instructions. The libraries are diluted to 25 pm, pooled and loaded on the Ion Chef to perform emulsion PCR and chip loading on 318 v2 chips. Sequencing was performed on ION PGM, using the HI-Q view Chef kit, according to the manufacturer’s instructions. Data were processed using the Torrent Suite 5.12.3 (TS). The quality of sequencing output was first evaluated through the plugin Coverage Analysis on the TS. Only samples whose library’s uniformity and on-target values were at least 80% and with a medium coverage of 1500X were considered valid. SNV analysis was performed in duplicate: the first variant calling was generated by the Variant Caller plugin from the TS and the resulting VCF file was loaded in the Variant Effect Predictor Tool (Ensembl, Version GRCh37) for the variants annotation. To eliminate erroneous base calling, we set each variant coverage > 40X, a variant frequency on each sample > 2% and a quality value > 30. Variants within homopolymer (HP) longer than eight bases and with strand bias ≥ 80% were not reported. In the second analysis, the BAM files were automatically uploaded from the TS to the Ion Reporter Software (IR, version 5.6 to 5.16) and the variant calling was integrated into the analysis pipeline “Oncomine BRCA Research Somatic—318”. The results of both analyses were manually compared. Each variant was displayed on IGV (ver. 2.3.97). Synonymous variants were filtered out, while the remaining variants were classified into pathogenicity classes according to the Evidence-based Network for the Interpretation of Mutant Alleles (ENIGMA) consortium guidelines (https://enigmaconsortium.org/). Our assay could not reliably detect large intragenic rearrangements.

### Germline testing

Two EDTA tubes of peripheral blood samples were collected from each patient who performed genetic counselling and was eligible for germline testing, either for targeted sequencing of tumour-detected pathogenic/likely pathogenic variants or for the analysis of large genomic rearrangements in patients with no actionable variants detected at tumour testing. Whole blood DNA was isolated through the MagCore^®^ Super automatic workstation with the MagCore^®^ Genomic DNA Whole Blood Kit (Diatech LabLine SRL, Jesi, Italy). Targeted Sanger sequencing of tumour-detected *BRCA1/2* PVs was performed on purified PCR products by using BigDye^®^ Terminator v.3.1 Cycle Sequencing kit (Thermo Fisher Scientific, Inc.) and run on 3730Xl DNA Analyzer (Applied Biosystems; Thermo Fisher Scientific, Inc.), after purification with Agencourt CleanSeq^®^-Beckman Coulter. Sequences were analysed by Mutation Surveyor^®^ Software (v5.0.1; SoftGenetics, LLC., State College, PA, USA). Targeted sequencing results were confirmed on both blood aliquots collected from each patient. Variants of uncertain clinical significance identified at tumour testing were not systematically investigated at the germline level. Eligible probands, who resulted negative at tumour testing with the Oncomine BRCA assay, were analysed for large deletions and duplications of *BRCA1* and *BRCA2* on blood DNA with the SALSA MLPA kits P045 *BRCA2*/*CHEK2* and P002 *BRCA1* probe mix (MRC-Holland, Amsterdam, the Netherlands), following the manufacturer’s instructions. MLPA products were run on the 3730Xl DNA Analyzer (Applied Biosystems; Thermo Fisher Scientific, Inc.) with the Gene Mapper Module (Applied Biosystems; Thermo Fisher Scientific, Inc.). The results were analysed through the Gene Marker Software v2.7.0 (SoftGenetics, LLC, State College, PA, USA).

### Assessment of homopolymeric regions and statistical analysis

Based on previous observations on the performance at homopolymers of ion semiconductor sequencing techniques, we focussed our analysis on stretches of six or more identical bases since the calling accuracy has been consistently shown to dramatically drop beyond this length^15,17,25,28,30^. We thus selected all 29 homopolymeric regions exceeding five repetitions to be analysed within the coding regions of both genes, including five in *BRCA1* and 24 in *BRCA2*. Since truncating variants beyond codon 3326 of *BRCA2* are not classified as high-risk variants, homopolymers downstream of the residue c.9976 of *BRCA2* were not included in the analysis (Table 2).

**Table 2:** List and genomic coordinates of all *BRCA1/2* homopolymeric regions longer than five repetitions.

Gene	Genomic coordinates GRCh37 (hg19)	Coding sequence	Lenght (bp)	Repeated base
*BRCA1* (NM_007294.3)	17:41,256,251–41,256,256	c.324_329	6	A
	17:41,247,865–41,247,870	c.663_668	6	A
	17:41,246,532–41,246,538	c.1010_1016	7	A
	17:41,245,587–41,245,594	c.1954_1961	8	A
	17:41,244,219–41,244,224	c.3324_3329	6	A
*BRCA2* (NM_000059.3)	13:32,890,628–32,890,633	c.31_36	6	T
	13:32,906,566–32,906,571	c.951_956	6	A
	13:32,906,603–32,906,608	c.988_994	7	A
	13:32,907,203–32,907,208	c.1588_1593	6	A
	13:32,907,421–32,907,428	c.1806_1813	8	A
	13:32,910,662–32,910,667	c.2170_2175	6	A
	13:32,911,074–32,911,080	c.2582_2588	7	A
	13:32,911,322–32,911,327	c.2830_2835	6	A
	13:32,911,443–32,911,449	c.2951_2957	7	A
	13:32,912,346–32,912,352	c.3854_3860	7	A
	13:32,912,656–32,912,661	c.4164_4169	6	T
	13:32,912,771–32,912,776	c.4279_4284	6	T
	13:32,913,080–32,913,085	c.4588_4593	6	A
	13:32,913,559–32,913,565	c.5067_5073	7	A
	13:32,913,784–32,913,789	c.5292_5297	6	A
	13:32,913,837–32,913,843	c.5345_5351	7	A
	13:32,914,070–32,914,075	c.5578_5583	6	A
	13:32,914,860–32,914,865	c.6368_6373	6	A
	13:32,929,162–32,929,167	c.7172_7177	6	A
	13:32,937,355–32,937,360	c.8016_8021	6	A
	13:32,953,633–32,953,639	c.8934_8940	7	A
	13:32,954,023–32,954,030	c.9090_9097	8	A
	13:32,954,273–32,954,279	c.9247_9253	7	A
	13:32,972,590–32,972,595	c.9940_9945	6	A

To overcome the limitations of the ION reporter software, which filters out most indels at homopolymeric regions, we manually visualised the BAM alignment files of the 222 patients at the 29 regions with the IGV software (ver. 2.3.97). The median depth of coverage of the regions of interest ranged from 1045 to 6989X. Each sample showed a variable frequency of sequence alterations (both insertions and deletions) at each region. We estimated the variant allele frequency (VAF) of insertions and deletions (indels) by calculating the ratios of the maximum inserted or deleted reads over the total reads at each homopolymer (Suppl. Table 1).

Since the VAF of indels at homopolymeric regions has, in general, a left-skewed distribution, we employed a modified version of the Cancer Outlier Profile Analysis (COPA) approach^31^, which consists in scaling the above-cited to a normal distribution and subsequently calculating the outliers that exceeded the mean + 3 median-adjusted deviations (μ + 3σ) threshold.

To validate the outliers, for each of the 29 regions, we further defined a threshold based on the normalized distributions (either for percentage of insertion or deletion) of a control population. Since both in ovarian and other BRCA-associated cancers the predominant second hit is most often represented by loss of heterozygosity (LOH), while a second point mutation is an extremely rare event^32–34^, we used as control population a cohort of 46 patients in which a non-homopolymeric PV (either somatic or germline) had been already identified.

To avoid potential selection bias, which would affect the estimated frequency of pathogenic variants occurring at homopolymeric regions in our cohort, we excluded from the analysis the 19 patients who underwent germline testing before tumour testing. This group also included two patients with germline-confirmed homopolymeric PVs who resulted negative at tumour testing.

Therefore, we applied the (μ + 3σ) thresholds estimated on the control population to the normalized distributions of the study cohort, composed of 157 individuals with no evidence of pathogenic variants at tumour testing with ION Torrent. In addition, according with filtering criteria used in a previous study, which focused on germline variants^29^, we considered only homopolymeric indels with an absolute VAF above 15% and in any case higher than the maximum value of the control population at each homopolymeric region. Lastly, regions with a total read count of less than 100 were excluded from the analysis.

Targeted Sanger sequencing was performed on tumour DNA to confirm the occurrence of outlier homopolymeric indels selected by using the defined thresholds.

## Supplementary Information

Supplementary Table 1.

## Data Availability

The datasets analysed during the current study are available in the NCBI SRA repository under the accession number PRJNA940102, https://dataview.ncbi.nlm.nih.gov/object/PRJNA940102 (reviewer link available at https://dataview.ncbi.nlm.nih.gov/object/PRJNA940102?reviewer=bva27dv8e88uglvdv5545vi1a1).
